# Substrate recognition and function of the R2TP complex in response to cellular stress

**DOI:** 10.3389/fgene.2015.00069

**Published:** 2015-02-25

**Authors:** Patrick von Morgen, Zuzana Hořejší, Libor Macurek

**Affiliations:** ^1^Department of Cancer Cell Biology, Institute of Molecular Genetics, Academy of Sciences of the Czech Republic, PragueCzech Republic; ^2^DNA Damage Response Laboratory, London Research Institute, LondonUK

**Keywords:** R2TP complex, protein folding, DNA damage response, cellular stress, cancer

## Abstract

The R2TP complex is a HSP90 co-chaperone, which consists of four subunits: PIH1D1, RPAP3, RUVBL1, and RUVBL2. It is involved in the assembly of large protein or protein–RNA complexes such as RNA polymerase, small nucleolar ribonucleoproteins (snoRNPs), phosphatidylinositol 3 kinase-related kinases (PIKKs), and their complexes. While RPAP3 has a HSP90 binding domain and the RUVBLs comprise ATPase activities important for R2TP functions, PIH1D1 contains a PIH-N domain that specifically recognizes phosphorylated substrates of the R2TP complex. In this review we provide an overview of the current knowledge of the R2TP complex with the focus on the recently identified structural and mechanistic features of the R2TP complex functions. We also discuss the way R2TP regulates cellular response to stress caused by low levels of nutrients or by DNA damage and its possible exploitation as a target for anti-cancer therapy.

## HSP90 AND ITS CO-CHAPERONES

Chaperones are proteins involved in protein folding and protein-complex assembly or disassembly (Macario and Conway de Macario, 2005). Heat shock protein 90 (HSP90) is an abundantly expressed chaperone implicated in a wide range of cellular processes, including cell signaling, protein degradation, genome maintenance and assembly of transcriptional and translational machineries (Prodromou et al., 1997; Li et al., 2012; Saibil, 2013). HSP90 client proteins are often in near-native state and HSP90 is involved in the late stages of their folding (Jakob et al., 1995). HSP90 acts as a dimer and its substrate specificity and activity are given by its co-chaperones (Ali et al., 2006; Li et al., 2012). Although most of the well-known HSP90 co-chaperones are small single-molecule proteins (CDC37, p23, SGT1, AHA1), recent work has shown that HSP90 co-chaperones can be multi-protein complexes themselves, such as the R2TP complex (Zhao et al., 2005; Li et al., 2012).

## R2TP COMPLEX

**FIGURE 1:**
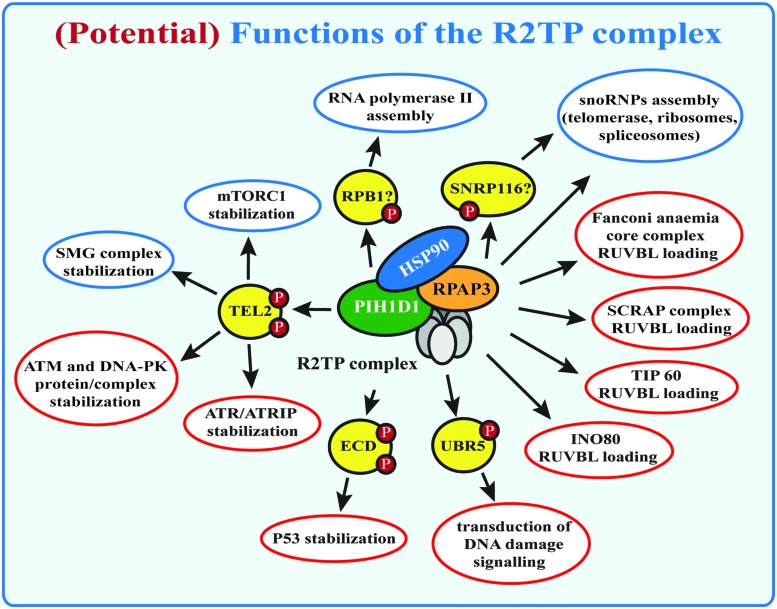
**(Potential) functions of the R2TP complex.** Known functions of the R2TP complex are indicated in blue, potential R2TP functions are indicated in red. SMG1 complex and mTOR stabilization by the R2TP complex requires binding of PIH1D1 with the PIH-N domain to TEL2. Other potential PIH-N domain binding proteins are indicated, but their role for R2TP complex function remains to be studied.

The R2TP complex was discovered in budding yeasts as an Hsp90-interacting protein-complex (Zhao et al., 2005). It is involved in the assembly of large number of multi-subunit complexes: (a) the small nucleolar ribonucleoproteins (snoRNPs), which are essential for biogenesis of ribosomes, spliceosomes, and tRNAs (Zhao et al., 2008; McKeegan et al., 2009); (b) RNA polymerase II (Boulon et al., 2010) and (c) phosphatidylinositol 3-kinase-related kinases (PIKKs) and their complexes (Horejsi et al., 2010) that are involved in DNA damage signaling (ATM, ATR, DNA-PKcs), transcription regulation (TRRAP), nonsense mediated mRNA decay (SMG1), and nutrient signaling (mTOR; Izumi et al., 2012; **Figure 1**). Although the R2TP complex has become recently focus of many studies, the exact function and the molecular mechanism of its action is still not clear.

## COMPONENTS OF THE R2TP COMPLEX

**FIGURE 2:**
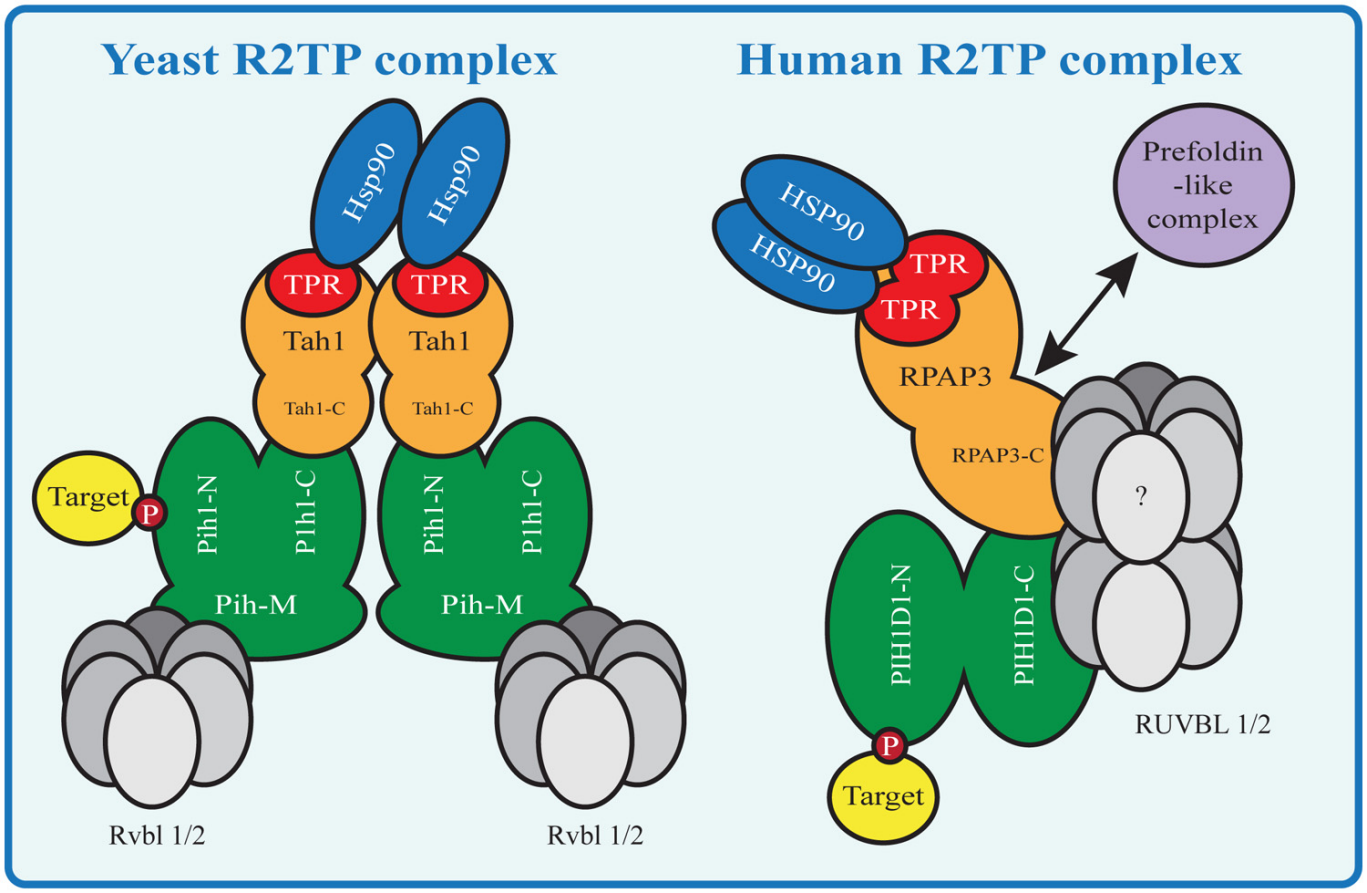
**HSP90-R2TP complex in yeast and mammals.** Proteins homologous between Yeast and mammals are shown in the same color. N indicates the N-terminal domain, C the C-terminal domain and M the middle domain of a protein. Yeast Tah1 contains one TPR domain and therefore two Tah1 molecules are needed to bind the Hsp90 dimer. Tah1 binds Pih1, which in turn binds R2TP target substrates with its N-terminal domain and the Rvbl proteins with its middle domain. In contrast the mammalian R2TP complex binds the HSP90 dimer with one RPAP3 molecule containing two TPR domains. The RPAP3 C-terminal domain connects directly or indirectly with the other components of the complex, namely PIH1D1 the RUVBLs and the prefoldin-like complex components (absent in yeast R2TP).

The R2TP complex is highly conserved from yeast to mammals and consists of four subunits: PIH1D1, RPAP3, RUVBL1 and RUVBL2, known under diverse names (**Table 1**; Zhao et al., 2005). The complex also associates with prefoldin and prefoldin-like proteins PFDN2, PFDN6, UXT, WDR92, URI and PDRG1, which form so called prefoldin-like complex, also implied in protein-complex assembly (Cloutier et al., 2009; Cloutier and Coulombe, 2010; **Figure 2**).

**Table 1:** Alternative names for the components of the R2TP complex.

	Mammals	Yeast
PIH1D1	NOP17	Nop17, Pih1
RPAP3	hSPAGH	Tah1
RUVBL1	Pontin, RVB1, TIP49A, TAP54α, ECP-54, TIH1, p50	Rvb1
RUVBL2	Reptin, RVB2, TIP49B, TAP54β, ECP-51, TIH2, p47	Rvb2

RUVBL1 and RUVBL2 belong to AAA+ ATPases family (ATPases associated with a variety of cellular activities) and are essential for viability in all so far examined model organisms (*Saccharomyces cerevisiae*, *Drosophila melanogaster*, and *Caenorhabditis elegans*; Nano and Houry, 2013). The AAA+ ATPases are characterized by presence of an AAA+ domain containing Walker A and B motifs, sensor domains 1 and 2 and an arginine finger (Patel and Latterich, 1998; Neuwald et al., 1999; Jha and Dutta, 2009). The Walker A motif binds ATP while the Walker B motif is involved in ATP hydrolysis, providing together ATPase activity. The sensor domains detect whether the ATPase is bound to ATP or ADP and the arginine finger either affects ATP hydrolysis or converts ATP hydrolysis into a mechanical output (Guenther et al., 1997; Ogura et al., 2004). Although *in vitro* data show no or limited ATPase activity of RUVBL1 and RUVBL2 (Grigoletto et al., 2011), some *in vivo* functions are impaired after mutation of the Walker A or B motifs: for example mutations of the yeast proteins comprising the ATPase activity result in serious growth defects while inactivation of the mammalian RUVBL1/2 activity decreases activation of mTOR and stability of the telomerase component TERC (Jonsson et al., 2004; Venteicher et al., 2008; Kim et al., 2013). It is therefore highly probable that the ATPase activity of RUVBL1/2 is important for at least part of their functions and that they might need other proteins, absent in the *in vitro* assays, that promote the ATPase activity. The purified proteins also exhibit a weak helicase activity (Huen et al., 2010).

The crystal structures of RUVBL1 revealed three structural subdomains – N terminal and C terminal subdomains form the AAA+ domain, while a flexible middle domain (also called the insertion domain), is involved in DNA or RNA binding. The insertion domain is located outside the core of the protein and is specific for RUVBL1/2 but not for other members of the AAA+ ATPases family and its deletion in both RUVBL1/2 increased their ATPase and helicase activity, indicating an auto-inhibitory function of this domain (Matias et al., 2006; Niewiarowski et al., 2010; Petukhov et al., 2012).

AAA+ ATPases often constitute hexamers (Smith et al., 2006) and accordingly, RUVBL1 and RUVBL2 form homo or hetero hexamers and/or double-hexameric structures – dodecamers. The crystal structure of a RUVBL1 monohexamer reveals a strong ADP binding by the AAA+ domain, which could explain its very low ATPase activity and indicating that the monohexamer is possibly not physiologically relevant (Matias et al., 2006). Yeast Rvb1 and Rvb2 incubated together form a hexameric ring, observed by electron microscopy and scanning transmission electron microscopy. Together the proteins have higher ATPase and helicase activity compared with the separate proteins (Gribun et al., 2008). Many studies also show formation of a dodecamer, consisting of both RUVBL1 and RUVBL2 (Puri et al., 2007; Torreira et al., 2008; Niewiarowski et al., 2010; Gorynia et al., 2011). Antibody labeling of Rvb2 in the yeast complex revealed that only one of the two rings contained this protein, arguing for two monomeric hexameres (Torreira et al., 2008). The crystal structure and mass spectrometry analysis of the human RUBVL1/2 complex supports formation of a dodecamer composed from two heterogenic hexameres (Niewiarowski et al., 2010; Gorynia et al., 2011). In the dodecameric complex, ATPase activity of both proteins is required to catalyze the ATP reaction (Puri et al., 2007) and depending on the arrangement of the insertion domain, the RUVBL complex forms a compact or a stretched confirmation (Lopez-Perrote et al., 2012). Interestingly, apart from a 3:3 ratio of RUVBL1/2, hexameres with different stoichiometry of RUVBL1/2 were also detected (Niewiarowski et al., 2010). The different conformations could represent the diverse range of functions these proteins play *in vivo*: one conformation could be important for helicase activity, while another one may be involved in protein-complex assembly (Torreira et al., 2008). Moreover, the independent and sometimes even opposing effects of RUVBL1/2 on transcription suggest that the ATPases can act independently on each other (Gallant, 2007).

RUVBL1 and RUVBL2 take part in many cellular processes: they are components of chromatin remodeling complexes TIP60, SWR/SRCAP, and INO80 and very recently they have been reported to interact with the Fanconi anemia core complex, which is involved in DNA inter-strand crosslink repair (Rosenbaum et al., 2013; Rajendra et al., 2014). RUVBL1/2 also influence transcription, play a role in assembly of the mitotic spindle and telomerase complex and (as a part of the R2TP complex) are involved in RNA polymerase II assembly, PIKK complex formation and snoRNPs biogenesis (Nano and Houry, 2013). The exact role of RUVBL1 and RUVBL2 in these processes remains to be determined. Published data suggest that their main function may be assembly or activation of the complexes in which they are contained. This theory supports the fact, that the protein level of Rvb1 and Rvb2 in yeasts is low compared to the abundance of the complexes in which they are involved and therefore only associate with these complexes transiently (Gallant, 2007; Nano and Houry, 2013).

RPAP3 and PIH1D1 are subunits specific exclusively for the R2TP complex. The yeast RPAP3 homolog Tah1 interacts with Pih1 via its C-terminal part and contains one tetratricopeptide (TPR) domain (Zhao et al., 2005; Millson et al., 2008). TPR domains are known to facilitate interactions with HSP70 or HSP90 and are implicated in other protein–protein interactions (Smith, 2004). The Tah1 TPR domain is involved in binding of Hsp90 but not of Hsp70 in stressed stationary cells and is not essential for Hsp90 recruitment to the R2TP complex (Millson et al., 2008; Zhao et al., 2008). While mutation of the Tah1 TPR domain decreased Tah1-Hsp90 interaction, it did not affect Tah1-Pih1 interaction. Tah1 forms a dimer, which binds both Hsp90 proteins present in the Hsp90 dimer, preventing its simultaneous binding to other co-chaperones (Pal et al., 2014). The drosophila RPAP3 homolog Spagh contains one TPR domain that binds both Hsp70 and Hsp90 and stimulates their activities (Benbahouche Nel et al., 2014). The presence of two TPR domains in human RPAP3 and its strong interaction with HSP90 and HSP70 indicate that RPAP3 couples the R2TP complex with HSP90 and possibly with other chaperones (Smith, 2004; Chagot et al., 2014). Crystallographic analysis of RPAP3 shows an interaction between both TPR domains and HSP90 peptides containing the conserved MEEVD sequence. Abolishing HSP90 binding requires mutation of both TPR domains, suggesting that the HSP90 dimer is bound by one RPAP3 molecule (Pal et al., 2014). RPAP3 is expressed in three isoforms: isoform 1 (and possibly isoform 3) interacts with PIH1D1 and is required for its stabilization, isoform 2 lacks an in-frame exon coding for 34 amino acids, present in the other two isoforms, and does not interact with PIH1D1, and therefore it may antagonize the R2TP complex activity. The C-terminal part of RPAP3 binds (independently on the TPR domains) a subunit of the prefoldin-like complex WDR92 and thus may mediate the interaction between R2TP and prefoldin-like complex (Itsuki et al., 2008; Back et al., 2013).

In yeast, Pih1 plays a central role in the R2TP complex by directly binding the other complex components (Kakihara and Houry, 2012). It has been proposed that Hsp90 and Tah1 stabilize the otherwise unstable Pih1 (Paci et al., 2012). Similarly to Tah1, Pih1 has Hsp90 binding properties, although the interaction is relatively weak, and the C-terminal domain of Pih1 binds the C-terminal domain of Tah1 (Zhao et al., 2008; Eckert et al., 2010; Paci et al., 2012; Back et al., 2013; Pal et al., 2014). In addition, Pih1 interacts with Rvb1 and Rvb2 by its middle domain. In mammals, RPAP3 and HSP90 also act together to stabilize PIH1D1 and PIH1D1 possibly connects the components of the R2TP complex (Zhao et al., 2008; Paci et al., 2012). While the middle domain is not present in mammalian PIH1D1, the C-terminal part ranging from amino acid 250 to 290 mediates the interaction with the rest of the R2TP complex, although it is not clear which of the R2TP complex components binds to the C-terminal part directly (Horejsi et al., 2014).

Two recently published papers identified a novel PIH1D1 phospho-peptide binding domain (PIH-N domain), distinct from any so far known phospho-binding-domains. The domain is capable of binding PIH1D1 interaction partner TEL2 independently of the rest of the complex. The PIH-N domain structures of the mouse and the human proteins are very similar. Both studies identified a basic patch within the N-terminal domain of PIH1D1, which is responsible for binding to TEL2 peptide containing a phosphorylated acidic DpSDD sequence. The crystal structure of the human PIH1D1 revealed hydrogen bonds essential for the binding between lysine 57, lysine 64, and arginine 168 of PIH1D1 (lysine 57, 64, and 133 in mouse) and the phosphorylated serine and aspartates of the DpSDD motif. Mutations of these PIH1D1 residues abolished TEL2 peptide binding (Horejsi et al., 2014; Pal et al., 2014). Lysine 57 and lysine 64 are evolutionary conserved and accordingly, mutation of lysine 64 in PIH1D1 abolished interaction with TEL2. Mass spectrometry and pull-down experiments revealed that PIH-N domain interacts directly in a phosphorylation-dependent manner with novel PIH1D1 interacting partners ECD, SNRP116, and UBR5. The mass spectrometry results also suggest that interaction between PIH1D1 and the main subunit of RNA polymerase II RPB1 is phosphorylation-dependent and that lysine 64 is essential for the interaction. Nevertheless, it is not clear whether the interaction with RPB1 is direct or mediated by another factor (Horejsi et al., 2014). These data indicate that PIH-N domain recognizes and recruits specific substrates to the R2TP during assembly of PIKKs, snoRNPs and RNA polymerase II and possibly of other complexes.

A phospho-binding domain similar to the PIH-N is probably also present in PIH1D1 ortholog Kintoun, which is involved in the cytoplasmatic assembly of dyneins, required for cilia motility (Omran et al., 2008). The positively charged lysines important for the PIH-N binding are substituted with positively charged arginines in Kintoun and it is capable of binding the phosphorylated TEL2 peptide with weak affinity (Horejsi et al., 2014; Pal et al., 2014). Since Kintoun interacts with DYX1C1, a TPR domain-containing protein involved in dynein assembly, it could function as a co-chaperone in a similar way as PIH1D1 in the R2TP complex (Tarkar et al., 2013).

## ASSEMBLY OF snoRNPs

SnoRNPs are RNA-protein complexes essential for biogenesis of ribosomes, spliceosomes, and telomerase (Smith and Steitz, 1997). The two major groups of snoRNPs are the box C/D snoRNPs (involved in processing of the pre-rRNA by 2^′^-*O*-methylation) and the H/ACA snoRNPs (involved in pseudouridylation of the pre-rRNA; Kiss-Laszlo et al., 1996; Tycowski et al., 1996). Assembly of snoRNPs is a complicated process, which requires a number of assembly factors.

The box C/D snoRNPs consist of box C/D snoRNAs and four core protiens: Snu13, Nop1, Nop56, and Nop58. Tah1 depletion in yeast led to decreased stability of box C/D snoRNAs and this effect was apparent only in stressed cells in stationary phase, possibly because Tah1 and Hsp90 are required for Pih1 stabilization under these conditions (Zhao et al., 2008). Rvb2 and Pih1 depletion led to a temperature sensitive phenotype and a disturbance in the accumulation and localization of both box C/D and H/ACA snoRNPs (Newman et al., 2000; Boulon et al., 2008). A synthetic genetic array, a method that identifies genes involved in the same pathway or complex, implicated genetic interactions of both Pih1 and Rvbs with Nop58p (Zhao et al., 2008). Pih1 directly binds the box C/D core protein Nop58 via Nop58 C-terminus (Gonzales et al., 2005; Kakihara et al., 2014) and the interaction is stronger in the absence of RNA. Binding of ADP, ATP, and ATPgS by Rvb1/2 (but not ATP hydrolysis) leads to dissociation of the R2TP complex itself and also releases R2TP from Nop58 C-terminal domain. Since the Walker A and Walker B motifs of Rvb1/2 are essential for yeast C/D snoRNA accumulation, the ATPase activity must play a role at a different step of the snoRNPs assembly (King et al., 2001; McKeegan et al., 2007, 2009; Boulon et al., 2008). Given that the R2TP complex interacts with the earliest stage of the snoRNP complex, which contains only the protein components, it is involved in the early phase of the box C/D snoRNP biogenesis. The mammalian RUVBL1 and RUVBL2 bind box C/D snoRNAs and all components of the R2TP complex are essential for their assembly (Newman et al., 2000; Boulon et al., 2008). Similarly to yeast Pih1, human PIH1D1 binds NOP58 and a snoRNP assembly factor NUFIP. A recent proteomic analysis revealed that RUVBL1 and RUVBL2 are components of various maturation stages of human pre-snoRNPs and are released at the final stage of snoRNP maturation. In accordance with the data from yeast experiments, PIH1D1 and RPAP3 were not detected in any stage of pre-snoRNPs, which raises the possibility that PIH1D1 and RPAP3 may in ATP dependent manner load RUVBL1/2 to the first RNA-free stage of snoRNP formation and do not take part in the latter stages of the assembly (Bizarro et al., 2014).

At least two assembly factors – Shq1 and Naf1 – are important for biogenesis of yeast H/ACA snoRNPs (Machado-Pinilla et al., 2012). Shq1 binds a component of the H/ACA snoRNP complex Nap57 and prevents the binding of Naf1 to the other snoRNP components (Yang et al., 2002; Grozdanov et al., 2009). Accordingly, the release of human SHQ1 from NAP57 is required for H/ACA snoRNP assembly. Cytosolic extracts from Hela cells were able to remove SHQ1 from NAP57 in an ATP independent manner and SHQ1 removal was inhibited by addition of antibodies directed at the R2TP component, but not by addition of HSP90 inhibitors. NAP57 directly binds PIH1D1 *in vitro* and interaction of its unstructured C-terminal part with RUVBL1/2 was essential for its disassociation from SHQ1. These experiments indicate that the R2TP complex takes action at the early stage of the H/ACA snoRNP assembly and is required for removal of inhibitors of H/ACA snoRNPs assembly from the H/ACA snoRNPs precursors. NAP57 lacks the DpSDD PIH-N domain consensus binding-motif, however, it contains phosphorylated acidic sequences that may mediate the interaction with the PIH-N domain. Since the purified R2TP complex was unable to release NAP57 from SHQ1, additional factors may be required for this reaction or for proper assembly of the R2TP complex itself (Machado-Pinilla et al., 2012).

## ASSEMBLY OF RNA POLYMERASE II

Eukaryotic cells contain three different RNA polymerases: (a) RNA polymerase I produces ribosomal RNA, (b) RNA polymerase II transcribes small nuclear RNAs and messenger RNAs, and (c) RNA polymerase III produces a range of small RNAs including the transfer RNAs. RNA polymerase II consists of 12 subunits of which Rpb1 and Rpb2 form the active cleft, while the other subunits are located further in the periphery of the complex (Cramer et al., 2008).

The R2TP complex together with the prefoldin-like complex interacts with RNA polymerase II and is involved in its assembly in the cytoplasm and in the transport of the assembled polymerase to the nucleus. Quantitative mass spectrometry analysis revealed that the polymerase is assembled in several steps, which include formation of two RNA polymerase II sub-complexes. The R2TP complex preferentially interacts with unassembled RPB1 and with the sub-complex containing RPB1. Depletion of RPAP3 and inhibition of HSP90 led to destabilization of RPB1 in the cytoplasm. Interestingly, RPAP3 also binds to RNA polymerase II subunit RPB5 independently on RPB1 and is required for its incorporation within the RNA polymerase II complex. RPB5 binds to the component of the human prefoldin-like complex URI, also involved in the assembly of RNA polymerases in the cytoplasm (Miron-Garcia et al., 2013). Thus, the prefoldin-like complex may be also involved in RPB5 assembly in the RNA polymerase II complex. It is highly possible that RPB1 is recruited to the R2TP complex via direct or indirect phosphorylation-dependent interaction with the PIH-N domain, because wild type but not mutated PIH-N domain binds phosphorylated RPB1 (Horejsi et al., 2014). RPB1 does not contain the DSDD motif, recognized by the PIH-N domain, but it contains other phosphorylated acidic Casein Kinse 2 (CK2) consensus sequences, which could bind to PIH-N. Alternatively, the interaction may be mediated by another factor binding to the PIH-N domain and to RPB1.

RNA polymerase I and III are also multi-subunit complexes. Since R2TP complex interacts with several of their subunits, it is highly possible that the R2TP complex is also involved in their assembly (Jeronimo et al., 2004, 2007; Boulon et al., 2012). In addition, PIH1D1 is directly involved in mTORC1-dependent rRNA transcription by RNA polymerase I (Zhai et al., 2012).

## ASSEMBLY OF PHOSPHATIDYLINOSITOL 3-KINASE-RELATED KINASES

The PIKK family consists of ATM, ATR, DNA-PKcs, mTOR, SMG1, and TRRAP. ATM, ATR, and DNA-PKcs are essential for DNA damage signaling (Zhou and Elledge, 2000; McKinnon, 2012); SMG1 regulates nonsense-mediated mRNA decay by the mRNA surveillance complex that removes mRNAs with premature stop codons (Yamashita et al., 2005); TRRAP is part of multiple acetyltransferase complexes and facilitates transcription by binding transcription factors like E2F and c-MYC (Grant et al., 1998; McMahon et al., 1998, 2000; Murr et al., 2007); and mTOR is a central player in cell metabolism and regulates processes like cell growth, autophagy, transcription, and actin organization in reaction to growth factor signaling and nutrient availability (Wullschleger et al., 2006). All PIKKs bind to HSP90 co-chaperone TEL2, which forms together with its interacting partners TTI1 and TTI2 so called TTT complex. TEL2 phosphorylated on serine 487 and 491 by CK2 binds to the PIH-N domain present in PIH1D1 and mutation of both serines disrupts its interaction with R2TP complex, but does not affect the binding of PIKKs and HSP90 (Horejsi et al., 2010). Therefore the TTT complex connects PIKKs to both HSP90 and R2TP complex independently of each other. Knock-out of TEL2 and knock-down of TTI1 ad TTI2 lead to depletion of PIKKs from cells (Horejsi et al., 2010; Hurov et al., 2010). Interestingly, HSP90 inhibition decreases levels of ATM and DNA-PKcs but does not affect levels of mTOR and SMG1 (Takai et al., 2007), while disruption of TEL2-R2TP binding affects stability of SMG1 and mTOR (Horejsi et al., 2010), but also of ATM and DNA-PKcs (Rao et al., 2014). At the same time depletion of RUVBL1 and RUVBL2 leads to decreased PIKKs levels (possibly by affecting both transcription of genes encoding RUVBL1/2 and protein stability) and reduces PIKK signaling (Izumi et al., 2012). It is therefore possible that HSP90 alone is important for proper folding of ATM and DNA-PKcs, while in complex with R2TP it mainly affects assembly of complexes of all PIKKs. As many subunits of large complexes become unstable if the complex formation is disrupted, it is possible that the instability caused by non-functional TEL2 or R2TP complex is not due to improper folding of the kinases themselves, but due to disruption of complexes in which they are involved. This is also supported by the fact that reduced levels of RUVBL1/2 impair formation of the mRNA surveillance complex, which contains SMG1 and mTORC1 complex (Izumi et al., 2010; Kim et al., 2013).

mTOR forms two distinct complexes: (a) mTORC1 is associated with Raptor and regulates protein synthesis, (b) mTORC2 is associated with Rictor and is involved in actin organization (Kim et al., 2002; Sarbassov et al., 2004). Study in mouse and human cells showed that knockdown of TEL2, TTI1, and RUVBL1/2 mediated by siRNA reduced mTOR activity several folds and led to disruption of mTOR dimer and impediment of the mTORC1 assembly (Kim et al., 2002; Sarbassov et al., 2004; Hoffman et al., 2010). The interaction between TEL2 and mTOR was dependent on the ATPase activity of RUVBL1 and RUVBL2 and was destabilized in the absence of ATP or glucose and glutamine starvation conditions (Kim et al., 2013). At the same time, destabilization of TEL2 and mTOR interaction led to decreased mTOR levels. Interestingly, low levels of glucose and glutamine led to decreased stability of other PIKKs as well, suggesting that nutrient signaling regulates also other cellular pathways by affecting the R2TP complex stability. The study in human and mouse cells focused only on members of the TTT complex and RUVBL1/2, but since TEL2 interacts with RUVBL1/2 through direct interaction with PIH1D1, the assembly of mTORC1 is regulated by CK2 (Fernandez-Saiz et al., 2013) and PIH1D1 directly binds to mTORC1, it is highly probable that the whole R2TP complex takes part in the mTORC1 assembly. This is also supported by the fact that the R2TP complex is involved in mTORC1 regulated rRNA transcription (Zhai et al., 2012).

ATR is activated in response to presence of single-stranded DNA, which is generated during repair of wide variety of DNA damage lesions and during replication stress. Upon activation, ATR forms a heterodimer with ATRIP and is recruited to the single-stranded DNA, coated by replication protein A (Nam and Cortez, 2011). Knock-down of TEL2 by siRNA firstly leads to decreased ATR activation and inhibits binding of the ATR/ATRIP heterodimer to DNA damage mediator protein TOPBP1 and later it leads to decreased levels of ATR and ATRIP (Rendtlew Danielsen et al., 2009). It is therefore possible that TEL2 interaction with the R2TP complex is required for formation of the ATR/ATRIP/TOPBP1 complex and that ATR and ATRIP become unstable if they cannot form the active complex.

## MOLECULAR MECHANISM OF R2TP FUNCTION AND ITS REGULATION

Although the work on snoRNPs assembly provides some clues about the mechanism of the R2TP complex function, most of it is still largely unknown. It seems that in mammals, PIH1D1 and RPAP3 are required at the early stages of complex formation, probably for loading of RUVBL1/2 to the assembled complex (Bizarro et al., 2014). RUVBL1/2 are required either to disassemble inhibitors of complex formation or are involved in some other steps of assembly and/or activation of the late stages of the assembled complexes (Nano and Houry, 2013). Given that RUVBL1/2 are known to be parts of many diverse cellular complexes, it is intriguing to hypothesize that the R2TP complex is involved in assembly of all these complexes.

The work on assembly of mTORC1 complex revealed that assembly of the R2TP complex itself is regulated by presence of nutrients and the complex becomes disassembled particularly in the absence of glucose and glutamine. The absence or presence of glucose and glutamine also regulates localization of the R2TP complex: in growing yeasts, the R2TP complex is localized in the nucleus and interacts with box C/D snoRNPs, while it relocalizes to the cytoplasm in poorly growing cells (Kakihara et al., 2014). These results show that nutrient signaling affects (via regulation of R2TP complex assembly and localization) various processes in the cells. Although the R2TP complex is also involved in regulation of DNA damage response, it is not known whether the DNA damage signaling affects localization or assembly of the R2TP complex.

## R2TP COMPLEX AND THE DNA DAMAGE RESPONSE

In order to protect genome integrity, cells are equipped with an extensive response mechanism that comes into play after DNA damage. Proper function of the DNA damage response pathways is essential for cancer avoidance (Bartkova et al., 2005).

The general mechanism of the DNA damage response consists of sensors, transducers, and effectors. The sensors detect the damaged DNA and activate PIKKs ATM, ATR and DNA-PKcs, which transmit the signal to effector proteins (Zhou and Elledge, 2000). Depending on the amount of the damage, the effector proteins (including transcription factor p53) arrest the cell cycle by activating the checkpoints and either repair the damaged DNA or (in case of too extensive damage) activate pathways leading to cellular senescence or apoptosis (Banin et al., 1998; Canman et al., 1998; Zhou and Elledge, 2000; Vousden and Lu, 2002).

The R2TP complex regulates the DNA damage response by affecting stability of all PIKKs involved in the DNA damage response and by regulation of ATR activity. In addition, mutation of the phospho-binding domain PIH-N caused decreased p53 activation following induction of DNA double strand breaks even in the presence of normal levels of ATM and DNA-PKcs, the two PIKKs responding to this type of DNA damage, suggesting that the R2TP complex regulates activity of ATM and DNA-PKcs or of other components of the DNA damage pathways (Horejsi et al., 2010). One of such components might be ECD, a protein that directly interacts with p53 and promotes its stabilization by inhibiting its binding to MDM2 (Zhang et al., 2006). ECD contains two DSDD motifs, which are both required for its direct phosphorylation-dependent interaction with the R2TP complex. Hypothetically, the R2TP complex may either guide the interaction between ECD and p53 or may be involved in the MDM2 disassociation from p53. The latter possibility is supported by the fact that HSP90 directly interacts with p53 and regulates its stability (Sasaki et al., 2007).

Another PIH1D1 phospho-binding partner involved in DNA damage is an E3 ubiquitin ligase UBR5, which regulates transduction of the DNA damage signaling to the effectors (Zhou and Elledge, 2000; Gudjonsson et al., 2012). The decreased p53 activity in the presence of mutated PIH1D1 could be due to disruption of the interaction between UBR5 and PIH1D1, although the mechanism is unclear.

The chromatin remodeling complexes TIP60, INO80, and SWI/SCRAP that contain RUVBL1/2 have been all implicated in regulation transcription, replication, recombination, and repair of DNA damage (Murr et al., 2006). The TIP60 complex possesses an acetyl-transferase activity, which is essential for accumulation of repair proteins at the site of the damaged DNA, activation of ATM (100) and removal of H2AX from the chromatin (Sun et al., 2005; Ikura et al., 2007). In addition, acetylation of p53 by TIP60 directs the p53 response toward induction of apoptosis. RUVBL1/2 are required for acetylation of at least some of the TIP60 targets (Ikura et al., 2007; Jha and Dutta, 2009).

INO80 is involved in repair of DNA double strand breaks by sliding nucleosomes along the DNA and their eviction at the site of the damage. Yeast Rvb1/2 bind to Ino80 subunit Arp5 in an ATP but not ATPase dependent manner and their loss leads to disassociation of Arp5 from the complex and to the loss of chromatin remodeling activity of the complex (Shen et al., 2003; Osakabe et al., 2014).

The yeast SWI complex, known as SRCAP complex in mammalian cells, remodels chromatin by catalyzing replacement of histone dimers H2A-H2B in nucleosomes by dimers containing the histone variant Htz1 in yeast or H2AZ in mammals. This process is essential for DNA damage signaling, although the role of RUVBL1/2 in it is not known (Niimi et al., 2012).

Very recently, it has been reported that RUVBL1/2 are associated with Fanconi anemia core complex. This complex is essential for recognition and repair of DNA cross-links. Knock-down of RUVBL1/2 shows similar phenotype as depletion of the Fanconi anemia core complex proteins and conditionally knock-out cells depleted from RUVBL1/2 have highly reduced levels of the Fanconi anemia core complex proteins (Rajendra et al., 2014).

The role of RUVBL1/2 in all the mentioned complexes is unknown, but in light of the recent findings about snoRNPs assembly and about the essential role of RUVBL1/2 in incorporation of Arp5 into the Ino80 complex, it is highly probable that RUVBL1/2 are necessary for assembly and activation of these complexes. It would be extremely interesting to know whether PIH1D1 and RPAP3 are involved in the early stages of their assembly by loading RUVBL1/2 onto these complexes.

## R2TP COMPLEX IN CANCER

Components of the R2TP complex are over-expressed in cancer: PIH1D1 levels are increased in several breast cancer cell lines (Kamano et al., 2013), RUVBL1/2 are over-expressed in liver and colon cancers (Huber et al., 2008; Jha and Dutta, 2009; Grigoletto et al., 2011) and their higher expression in cancer tissues is positively correlated with the expression of genes involved in metabolic processes activated by mTOR signaling (Kim et al., 2013). HSP90 levels are high in various cancer cell lines (Calderwood et al., 2006) and its activity is essential for variety of processes in cancer cells. It also plays a key role in the conformational maturation of oncogenic signaling proteins such as HER-2/Erb2, Akt, Raf-1, Bcr-Abl, and mutated p53. Multiple drugs that influence the substrates of R2TP or HSP90 chaperone are currently used or tested for the treatment of cancer. HSP90 became an attractive antineoplastic drug target, currently with 17 agents in different stages of clinical trials (Neckers and Workman, 2012) and inhibition of HSP90 reveals significant treatment effects *in vitro* in different types of cancers of unmet need such as the glioblastoma, lung, and pancreas cancer (Mayer et al., 2012; Choi et al., 2014; Pillai and Ramalingam, 2014; Wachsberger et al., 2014). Inhibition or depletion of HSP90 co-chaperones such as p23, Cdc37, and Aha1 further sensitizes cells to HSP90-targeted drugs (Neckers and Workman, 2012). Increased mTOR activity can promote tumor growth and mTOR inhibitors are already used in the clinic (Fruman and Rommel, 2014). CK2 is essential for cellular viability and progression of the cell cycle. It is required for tumorigenesis, but the exact mechanism is not clear (Trembley et al., 2010). Sites phosphorylated by CK2 form protein–protein interaction motifs, critical for regulation of DNA damage response pathways (Iles et al., 2007; Chapman and Jackson, 2008; Horejsi et al., 2010; Yata et al., 2012; Guerra et al., 2014; Horejsi et al., 2014) and for recognition of specific substrate by the R2TP complex. The relevance of CK2 as a molecular target in cancer has led to the development of CK2 inhibitors for clinical use, which are potent, highly specific and orally available, with known antitumor efficacy in breast, pancreatic and prostate xenograft mouse models (Cozza et al., 2013; Gyenis et al., 2013).

The R2TP complex specifically recognizes sites phosphorylated by CK2 and works in conjunction with HSP90 to assemble multi-subunit complexes involved in many cellular processes highly relevant to cancer, which makes it a promising target for new cancer drugs. Potential targets include the ATPase activity of RUVBL1 or RUVBL2 (Elkaim et al., 2012; Grigoletto et al., 2013) and PIH-N domain binding – inhibitors that block the PIH-N phospho-binding by mimicking the DpSDD motif should prevent function of the R2TP. The R2TP complex inhibitors could be of value in a clinical setting either to enhance HSP90, CK2, ATM and ATR inhibitors, to sensitize cells to cancer treatment based on induction of DNA damage (radiotherapy and some types of chemotherapy) or as an alternative to HSP90 inhibitors.

## FUTURE DIRECTIONS

**FIGURE 3:**
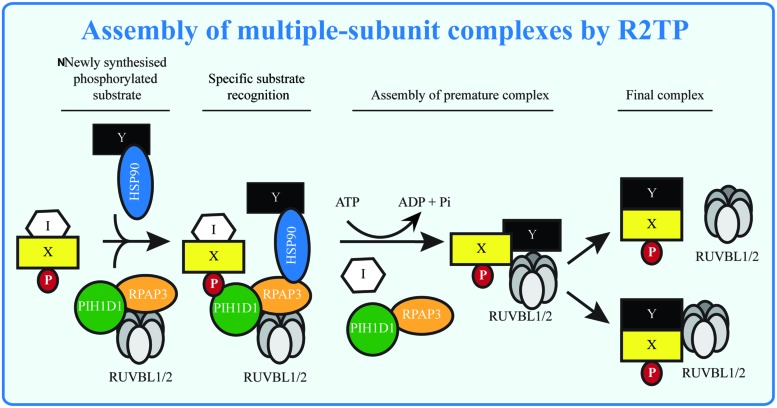
**Assembly of multiple-subunit complexes by R2TP.** The multiple-subunit complexes in mammalian cells are assembled in several steps: the early phase requires presence of R2TP complex, which may serve to remove assembly inhibitors (I) and/or to load RUVBL1/2 to the premature complexes in ATP dependent manner. RUVBL1/2 helps in the latter stage of assembly forming the mature complexes and either disassociate from or become part of the mature complexes. The substrate (X, Y) specificity is given by presence of phosphorylated sequence (P), recognized by PIH-N domain and possibly by specific interactions with other components of the R2TP complex.

The R2TP complex is implicated in the assembly of multiple cellular complexes (**Table 2**). The mechanisms by which the R2TP complex recognizes its substrates and exerts its function are still not completely understood. Strikingly, the experiments with snoRNPs assembly indicate that disruption of protein interactions and loading of RUVBL1/2 onto the premature complexes may be a general R2TP function (**Figure 3**). Indeed, NOP56 and NOP58, components of boxC/D snoRNPs, have homology to the unstructured tail of H/ACA snoRNP component NAP57, important for NAP57 disassociation from SHQ1 (Machado-Pinilla et al., 2012). The identification of the PIH-N domain helps us to understand the principle of recognition of R2TP substrate and could assist in the search for novel R2TP complex substrates. As the interaction between PIH-N and its substrate is phosphorylation-specific and the main kinase involved in the substrate phosphorylation seems to be CK2 (Horejsi et al., 2010, 2014), better understanding of its activation in response to different stimuli and/or identification of other kinases phosphorylating the substrate will lead to better understanding of regulation of R2TP function. Indeed, although CK2 has been regarded as a constitutive kinase not subject to regulation, recently published data show that binding of 5-diphosphoinositol pentakisphosphate (IP7) to CK2 enhances its phosphorylation of TEL2 and increases stability of DNA-PKcs and ATM after DNA damage (Rao et al., 2014).

**Table 2:** Confirmed and potential targets of the R2TP complex.

	Function		Direct interaction	
R2TP complex target	Target complex	R2TP complex	R2TP component	Target complex component
**Confirmed**
RNA polymerase II	Transcription	Assembly	?	?
snoRNPs	Biogenesis of ribosomes, spliceosomes, and telomerase			
boxC/D snoRNPs	2^′^-*O*-methylation of preRNA	Assembly	PIH1D1?	NOP58?
H/ACA snoRNPs	Pseudouridylation of prerRNA	Assembly	PIH1D1?	NAP57?
SMG1	Nonsense mediated mRNA decay	Assembly	PIH1D1	TEL2
mTORC1	Central regulator of cell metabolism	Assembly	PIH1D1	TEL2
ATM/ATR/DNA-PK	Kinases involved in DNA damage signaling	Possibly assembly	PIH1D1	TEL2
**Potential**
ECD/p53	Transcription factor involved in DNA damage response	possibly assembly	PIH1D1	ECD
UBR5	E3 ubiquitin ligase involved in DNA damage signaling	?	PIH1D1	UBR5
INO80	Chromatin remodeling complex, involved in repair of DNA double strand breaks	Possibly loading RUVBL1/2	?	?, RUVBL1/2
TIP 60	Chromatin remodeling complex, acetyl-transferase activity important for DNA damage response	Possibly loading RUVBL1/2	?	?, RUVBL1/2
SWI/SCRAP	Chromatin remodeling complex, DNA damage signaling	Possibly loading RUVBL1/2	?	?, RUVBL1/2
Fanconi anemia core complex	Recognition and repair of DNA crosslinks	Possibly loading RUVBL1/2	?	?, RUVBL1/2

One of the main functions of the R2TP complex could be regulation of cellular energy balance. The assembly of RNA polymerase II, mTORC1, ribosomes and spliceosomes (through the snoRNPS) is dependent on the R2TP complex. The presence of ATP increases the presence of higher order RUVBL complexes (McKeegan et al., 2009) and stimulates R2TP mediated assembly of mTOR and rRNA transcription (Kim et al., 2013). Therefore R2TP may work as a metabolic switch or master regulator by simultaneous influencing mTOR activity, protein synthesis and other cellular processes such as transcription and response to DNA damage.

Most of R2TPs functional mechanisms still remain elusive: how does R2TP assert its function on its substrates? What is the role of the prefoldin/like complexes associated with the R2TP complex? Are the differences in the structures reported for the RUVBL hexameres relevant for its function? What is the role of HSP90 in the R2TP complex? Is the PIH-N domain always involved in R2TP substrate recognition? Is PIH-N domain involved in regulating assembly processes? Is the R2TP complex generally involved in assembly of complexes containing RUVBL1/2? Answering these questions will allow us to start understanding of the molecular mechanisms of the function of this highly important complex. Also, more studies are required to evaluate the attractive possibility that the R2TP inhibitors are relevant in cancer treatment.

## Conflict of Interest Statement

The authors declare that the research was conducted in the absence of any commercial or financial relationships that could be construed as a potential conflict of interest.
